# How Neuroimaging Can Aid the Interpretation of Art

**DOI:** 10.3389/fnhum.2021.702473

**Published:** 2021-09-14

**Authors:** Ladislav Kesner, Petr Adámek, Dominika Grygarová

**Affiliations:** ^1^National Institute of Mental Health, Klecany, Czechia; ^2^Faculty of Arts, Masaryk University, Brno, Czechia; ^3^Third Faculty of Medicine, Charles University, Prague, Czechia

**Keywords:** gaze, eye contact, brain imaging, inference, art, interpretation

## Abstract

Cognitive neuroscience of art continues to be criticized for failing to provide interesting results about art itself. In particular, results of brain imaging experiments have not yet been utilized in interpretation of particular works of art. Here we revisit a recent study in which we explored the neuronal and behavioral response to painted portraits with a direct versus an averted gaze. We then demonstrate how fMRI results can be related to the art historical interpretation of a specific painting. The evidentiary status of neuroimaging data is not different from any other extra-pictorial facts that art historians uncover in their research and relate to their account of the significance of a work of art. They are not explanatory in a strong sense, yet they provide supportive evidence for the art writer’s inference about the intended meaning of a given work. We thus argue that brain imaging can assume an important role in the interpretation of particular art works.

## Introduction

Over the past two decades, the sister disciplines of the cognitive neuroscience of art and neuroesthetics have enjoyed growing recognition within the mind and brain sciences. The progress in these fields has not, however, yet translated into increasing acceptance in and synergy with humanities disciplines such as art history and theory or visual studies. Criticism and dismissal of neuroesthetics (Brown and Dissanayake, 2009, p. 44; Massey, 2009; Hyman, 2010, p. 182–185; Minissale, 2013, p. 112–113; Gopnik, 2012; Conway and Rehding, 2013; Bundgaard, 2015; Noë, 2015; Vassiliou, 2020) are far more common in the humanities than any positive appraisals. In their broad overview of the state of the field, Pearce et al. (2016) concede that neuroesthetics has continued to be criticized in the humanities because of its failure to produce interesting results about art itself. While some of the criticisms appear to be motivated by an *a priori* bias, and misrepresent the aims and procedures of recent neuroscientific studies of art (see, e.g., Rampley, 2017 for an example of misguided criticism), most of them offer salient points worth considering, which those working in this area would ignore at their peril. The brunt of the criticism is well summed up in philosopher Alva Noë’s statement that neuroscience is not a suitable technique for studying art, and that art “objects themselves…play almost no role in neuroscience (let alone in the neuroscience of art)” (Noë, 2015). Meanwhile, the view that neuroscience of art has nothing to offer when it comes to understanding specific art works continues to be asserted, as revealed in a recent statement: “But can aesthetics from below illuminate the appeal of the painting by Mondrian? The answer must be no. That a painting exhibits any of the appealing features I have just discussed is neither necessary nor sufficient for understanding it or evaluating it. The only discourse that can help us understand *this* Mondrian is the discourse of art history, of hermeneutics, of aesthetics from above” (Kubovy, 2019, p. 15). Perhaps just as disturbing as the explicit criticism is the fact that the potential of the cognitive neuroscience of art (and more broadly of experimental research) is barely mentioned in recent methodological and theoretical texts in art history and theory.

Importantly, the limits to the understanding of individual art works obtained from experimental brain research have been acknowledged also by those directly involved in this line of work. The question has been asked whether neuroimaging techniques can adequately deal with the interplay of cognitive, affective, personal, social, and cultural factors (Cela-Conde et al., 2011). It has been argued that “[n]euroscience is unlikely to address sociological or historical conceptions of art with any specificity…and unlikely to contribute much to cultural and sociological aspects of art” (Chatterjee, 2012, p. 299, 310), and in their overview of the field Chatterjee and Vartanian (2014) concede that “[n]euroscience methods do not easily address this level of textured meaning embedded within individual works of art.” One reason for this situation may be provided by the observation that most research in empirical aesthetics has disregarded the theoretical consequences of historical and contextualist approaches in the arts (Bullot and Reber, 2013). Critics from the humanities and contributions from within neuroesthetics seem to converge on the point that experimental brain research is not well equipped to offer useful evidence for the hermeneutical task of interpretation. At any rate, the question of “…what should we be asking of art with empirical brain research”? (Donald, 2006, p. 13) continues to be highly relevant. In this article, we revisit a study conducted recently by our team and present a case study to demonstrate that neuroimaging research can, in fact, be related to the task of interpreting and understanding a particular art work, thus making it directly relevant to the concerns of interpreters in the humanities.

## Direct Versus Averted Gaze in Painted Portraits

One broad topic that features prominently both in the mind and brain sciences and in the humanities concerns gaze and eye contact. The direct gaze plays a central role in social interaction and cognition (Argyle and Cook, 1976; George and Conty, 2008) and neuroimaging techniques have been extensively utilized to study the neurocognitive mechanisms of various aspects of eye contact and its role in social interaction and communication (Nummenmaa and Calder, 2009; Senju and Johnson, 2009; Hamilton, 2016). The advent of “second-person neuroscience” (Pfeiffer et al., 2013; Schilbach et al., 2013) ushered in a decade of intense exploration of eye contact effects, which have lately utilized hyperscanning methods. Gaze has similarly become an academic industry of its own in contemporary art theory and visual and film studies. Leaving aside the vast amount of critical theorizing inspired by psychoanalysis and feminist approaches, art historians have sought to understand the practice of visual artists who for centuries intuitively manipulated the direction of the gaze of the persons they depicted to imbue their work with distinct psychological effects. Mostly by employing subtle phenomenological analysis, art historians, for their part, have discussed manifold ways in which the direct gaze of the depicted subject engages the viewer and draws him/her into imaginative communicative interaction (Panofsky, 1953; Wollheim, 1987; Berger, 1994; Belting, 2009; Elkins and Fiorentini, 2020).

The dual importance of gaze and eye contact that bridges the cognitive sciences and the humanities has inspired our study. Consisting of separate fMRI and eye-tracking experiments, the study was designed to explore the neuronal and behavioral response to painted portraits with a direct versus an averted gaze. We sought to identify how the neural response to emotional expressive faces in paintings is modulated by the direction of a gaze. To explore the behavioral influence of the gaze direction of artistic portraits on the beholder’s eye movements and visual scanning, we supplemented the fMRI study with a separate eye-tracking experiment. The portraits were organized in duplets: each duplet contained two portraits by the same artist, the first portrait being classified as a “direct gaze” and the second one as an “averted gaze.” The subjects were instructed to observe the stimuli as if they were looking at paintings in gallery, no explicit evaluation task was involved. The study revealed that the portraits that established eye contact versus those with an averted gaze elicited increased activation in the lingual and inferior occipital gyri and the fusiform face area, in the dorsolateral prefrontal cortex, and in several of the areas involved in social cognitive processes, especially the theory of mind: the angular gyrus/temporo-parietal junction and the inferior frontal gyrus (Kesner et al., 2018). The same areas are typically activated in direct gaze contact in studies using more naturalistic stimuli (Senju and Johnson, 2009) and – recently – also live eye-to-eye contact (Cavallo et al., 2015; Kegel et al., 2020; Noah et al., 2020; Kelley et al., 2021). However, our set of portraits with a direct gaze did not activate the ventromedial prefrontal cortex, the amygdala, and the posterior superior temporal sulcus, which may indicate the subjects’ implicit awareness that they were not face to face with a living person.

The results of our study thus suggest that static and, in some cases, highly stylized depictions of human beings in artistic portraits elicit a pattern of brain activation that is similar to the experience of being observed by watchful intelligent beings. Our findings support the conclusion that the perception of a direct gaze in a portraits involved our subjects in implicit inferences of the painted subject’s mental states and emotions and primed them for a potential communicative act with the depicted persons – they perceived portraits not exactly like live people, but not like inanimate things either. The results were thus consistent with a model positing that the perception of figurative art depends on dynamic and fluctuating interaction between two interlinked sets of processes: socio-affective/cognitive processing, involved in person perception, and symbolic/aesthetic processing, concerned with the non-social aspects of an image (Kesner and Horáček, 2017). The findings from our supplementary eye-tracking experiment were consistent with our neuroimaging results and confirmed that participants spent more time viewing the depicted person’s eyes when viewing the direct gaze portraits than the averted ones. The heightened attention to a direct gaze observed here thus supports the neuroimaging findings and confirms that gaze is a crucial feature that drives face processing and social engagement. Let us now briefly consider how the results of this neuroimaging experiment can be related to an art-historical interpretation of a work of art.

## Activating Affordance in a Painted Gaze

**FIGURE 1:**
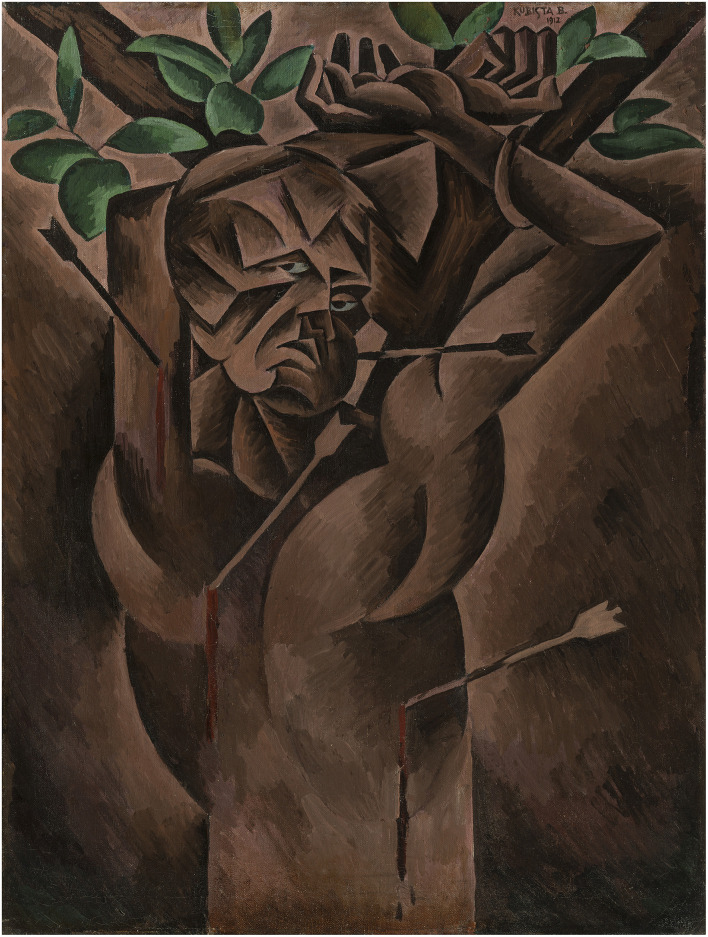
Bohumil Kubišta, Saint Sebastian, 1912. Oil on canvas, 98 × 75 cm. Collection of National Gallery, Prague.

Bohumil Kubišta’s *Saint Sebastian* of 1912 has been singled out as a key work of art in central European painting dating from the years before WWI (Srp et al., 2014, p. 252). Scholars agree that the painting should be seen as the artist’s imaginary self-portrait, the saint being a metaphor for the troublesome personal ordeal of the artist, who struggled with misunderstanding on the part of his audience and with poverty (Figure 1). All accounts of the work emphasize that it is a portrait of desperate pain and suffering. The emotional charge of the painting comes through in the words of the painter Jan Zrzavý, Kubišta’s friend: “The tormented face of the saint, the helplessness of his tied[bound] arms, his helplessness, the pitifulness of his naked body, the sadness of the bleak dark red reveal so suggestively, so deeply the human pain of the artist’s life” (Zrzavý, 1949). Other commentators have highlighted the painter’s ability to deploy the interposition of basic geometric forms – triangles, grooves, and small arcs – to render a facial expression (or “field lines”) that conveys “desperate pain and suffering” (Kubišta, 1940, p. 51). It has also been noted that if the face is perceived in isolation, the viewer all the more absorbs the unusually intense sadness being conveyed by the eyes. Various accounts thus concur that the meaning of the picture is directly tied to the emotional effect in produces in its spectator.

**FIGURE 2:**
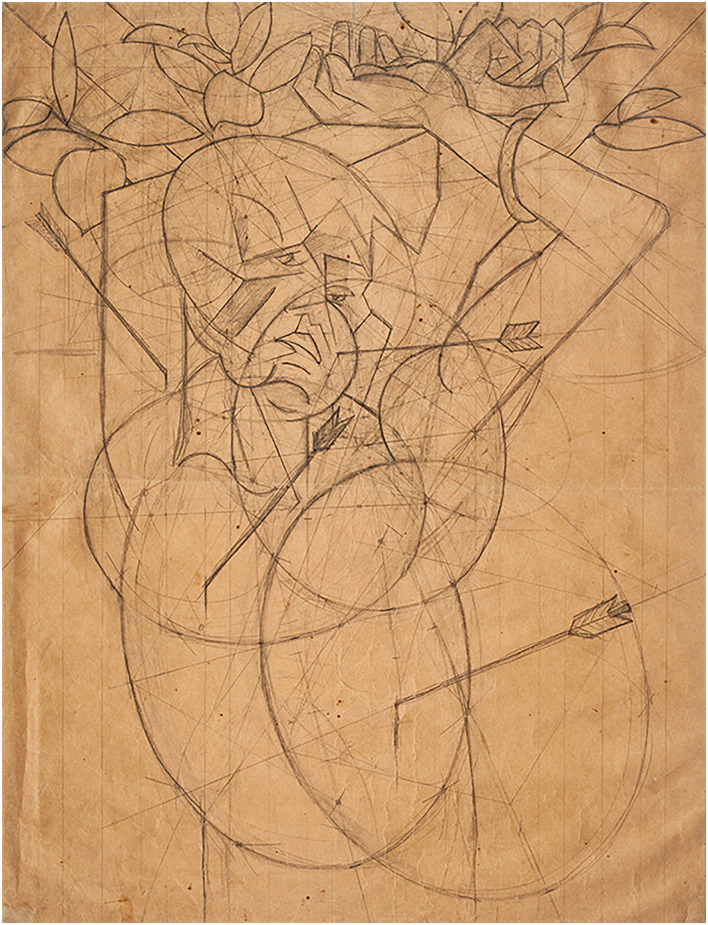
Bohumil Kubišta, Study for Saint Sebastian, 1912. Pencil on paper, 95 × 74 cm. Collection of National Gallery, Prague.

Another key feature emerges when the painting is juxtaposed with the preparatory drawing, in which the painter created an elaborate compositional structure that he later adapted for the oil version (Figure 2). Scholars agree that the saint’s head constitutes the compositional and ideographic center of the painting. What immediately stands out is the difference in the most prominent, attention-grabbing visual and affective salience of the two images, which is to say the difference in the way Kubišta rendered the gaze of the figures in the preparatory drawing and the painting. He changed the averted gaze of the figure in the drawing to a direct gaze in the painting, which decisively alters the expression and hence the effect of the two images on the viewer. While in the preparatory drawing, the averted gaze and a half-closed left eye result in the expression of silent endurance and resignation, in the oil painting the gaze is focused on the viewer, which imbues the face with an inquisitive, defiant directness, as if the saint (the artist) is appraising or challenging the viewer, thereby establishing communicative interaction with him/her. Thus, while both the drawing and the painting may share an overall theme and artistic intention, namely to present a visual metaphor of Kubišta’s own struggle with the world at large, the semantic difference in the affective affordance of the gaze/expression substantially modifies the meaning in each version (Kesner, 2016). Such conclusions are supported by vast amount of experimental evidence on the effects of direct gaze in social interaction and non-verbal communication (e.g., Ewing et al., 2010; Conty et al., 2016; Hietanen, 2018; Cañigueral and Hamilton, 2019), as well as phenomenological accounts of gaze perception (Heron, 1970; Stawarska, 2006) and observations on effects of direct gaze in paintings specifically (e.g., Wollheim, 1987; Huber, 2005).

Kubišta left no written testimony about the process of actually working on this canvas; however, given his intense preoccupation with the direction of the gaze of figures in his other portraits and self-portraits from this period, it is plausible to assume that the re-working of Sebastian’s gaze was not at all accidental. Rather, one can see it as a visible manifestation of his developing artistic intention, which fully envisaged the different effects the two distinct renderings would have on their viewers. The change in the gaze’s direction and the resulting change in the total expression signals a shift in Kubišta’s intention from an image of the Self as a more passive victim of circumstances and others’ will, to a Self who, self-consciously addressing and questioning the implied viewer, appears to resist and challenge his ordeal, which is embodied by the implied viewer. In other words, the different potential of the same affective affordance establishes a space in which, within an overall theme of metaphorical martyrdom, meaning fluctuates and is established through a particular viewer’s embodied understanding (Kesner, 2016). But how does the epistemology of the fMRI and eye-tracking experiments relate to such an art historical narrative?

## Relating Neuroimaging Results to an Art Historical Narrative

As in other domains of cognitive neuroscience, empirical research on aesthetic experience using neuroimaging has been struggling with the problem of reverse inference (Poldrack, 2006; Hutzler, 2014), an interpretive problem that arises when cognitive processes are inferred from the activation of a particular brain region. In fact, reverse inference may be said to be a particularly thorny issue for the cognitive neuroscience of art, given the fact that perceiving art works engages a plethora of brain areas (Vartanian and Skov, 2014), all of which have been known to be active in a number of cognitive and affective operations. It is worth pointing out that art history and criticism have been grappling with their own version of the “reverse inference” conundrum. As celebrated art historian Michael Baxandall pointed out, inferential criticism, which “entails the imaginative reconstruction of causes, particularly voluntary causes or intentions within situations” (Baxandall, 1979, 1985), is a major trope of art writing.

More broadly, art writing that focuses on interpreting a particular work of art typically involves a process known as “abductive inference” (Harman, 1965; Lombrozo, 2012), in which some visual features of the work, as described in the phenomenology of the art writer’s experience, are causally related to its creation. The account of the shift in the gaze direction in Kubišta’s painting, explained above, is a standard example of such an inference in art writing, where a salient visual/formal feature of the work is linked to the artist’s intention and hence to the meaning of the work. The best art interpretation and criticism (such as that of Max Dvořák, Leo Steinberg, Michael Fried, Lucy Lippard, Michael Baxandall – to offer but a few notable and otherwise disparate examples) emerge from such a pattern of inference, which ascribes a subjective account with the status of something objectively given.

Still, many art historians argue that perceptual and affective response – and by implication experimental approaches that are concerned with them – are irrelevant to the meaning of an art work (e.g., Ashton, 2011; Gopnik, 2012; Cronan, 2013; Noë, 2015). It is argued that experiential effects are different for each body and those differences need not be reconciled (Cronan, 2013, p. 26). Our case study suggests otherwise. First, it shows – contrary to such assertions – that the perceptual and affective response to salient social/affective affordances (in our case the direction of a gaze) do in fact play a central role in the meaning-making process. Whatever deeper meanings there are, mediated by various literary and other cultural associations the work possesses, they are grounded in and accessible through the beholder’s response, some aspects of which are open to empirical investigation. Second, in our case study, the psychological effect that is produced in the interpreter’s experience of the work is then linked to the artist’s intention and given a causal role in the genesis of the picture. The reality of this experience-based inference is then correlated with neuroimaging data from the group-level analysis of responses to the same aspect of the picture. These data provide evidence in support of the art writer’s interpretive inference about the picture. Such evidence is corroborated by contextualizing experimental data through relevant psychological findings on gaze effects, as noted above. The evidentiary status of neuroimaging data in our case is thus no different from any other extra-pictorial facts (e.g., biographical, technical, stylistic, historical, and contextual) that an art historian may uncover and relate to his/her observation of some salient visual feature in an image. It is not (and in principle cannot be) explanatory in a strong sense, yet it is used to substantiate the art writer’s inference about the intended meaning of a given work. An interpretation of a work of art cannot be “right” or “wrong” *per se*; however, an acceptable interpretation is one that is true to the facts and plausible (Carrier, 1991). Brain imaging can thus significantly contribute to the acceptable interpretation of particular works of art.

## Conclusion

There has recently been a shift in the conceptualization of neuroesthetics, which is increasingly accepted as the *cognitive neuroscience of aesthetic experience* (Pearce et al., 2016). This has some serious implications. If we accept the axiom – variously defended by many writers – that the meaning of any work of art is not a given and that it is rather established in and through the viewer’s experience (cf., e.g., Kemp, 1998; Kesner, 2006), then it logically follows that the cognitive neuroscience of art not only has a role to play in uncovering the mechanisms of aesthetic response and validating hypotheses about art perception, but can be productively employed in the quest to establish the meaning of particular work of art as well. Here we have presented one model of how this can be accomplished, but there are undoubtedly other options waiting to be explored. What this step requires is much closer interdisciplinary cooperation (such as has been increasingly evident in psychological experiments on art perception, cf., e.g., Commare et al., 2018; Reitstätter et al., 2020) in which art theorists and visual culture experts are directly involved in planning and designing experimental neuroimaging work.

## Data Availability Statement

The original contributions presented in the study are included in the article/supplementary material, further inquiries can be directed to the corresponding author.

## Author Contributions

LK drafted the first version of the manuscript. PA and DG contributed to writing. All authors contributed to the article and approved the submitted version.

## Conflict of Interest

The authors declare that the research was conducted in the absence of any commercial or financial relationships that could be construed as a potential conflict of interest.

## Publisher’s Note

All claims expressed in this article are solely those of the authors and do not necessarily represent those of their affiliated organizations, or those of the publisher, the editors and the reviewers. Any product that may be evaluated in this article, or claim that may be made by its manufacturer, is not guaranteed or endorsed by the publisher.
